# Sex differences in the association between prenatal exposure to maternal obesity and hippocampal volume in children

**DOI:** 10.1002/brb3.1522

**Published:** 2020-01-05

**Authors:** Jasmin M. Alves, Shan Luo, Ting Chow, Megan Herting, Anny H. Xiang, Kathleen A. Page

**Affiliations:** ^1^ Division of Endocrinology Department of Medicine Keck School of Medicine University of Southern California Los Angeles CA USA; ^2^ Diabetes and Obesity Research Institute Keck School of Medicine University of Southern California Los Angeles CA USA; ^3^ Department of Research and Evaluation Pasadena CA USA; ^4^ Department of Preventive Medicine University of Southern California Los Angeles CA USA

**Keywords:** childhood, hippocampal subfields, hippocampal volume, maternal obesity, sex differences

## Abstract

**Introduction:**

Animal studies have shown that male but not female offspring exposed to maternal obesity have abnormal hippocampal development. Similar sex differences were observed in animal models of developmental programming by prenatal stress or maternal diabetes. We aimed to translate this work into humans by examining sex‐specific effects of exposure to maternal obesity on hippocampal volume in children.

**Methods:**

Eighty‐eight children (37 boys and 51 girls) aged 7–11 years completed the study. Maternal prepregnancy body mass index (BMI) was obtained from electronic medical records. A high‐resolution anatomical scan was performed using a 3‐Tesla magnetic resonance imaging (MRI) scanner. Total hippocampal volume and hippocampal subfield volumes were analyzed using FreeSurfer 6.0. Linear regression was used to investigate sex differences in relationships between maternal prepregnancy BMI and child hippocampal volume.

**Results:**

Maternal prepregnancy BMI ranged from 19.0 to 50.4 kg/m^2^. We observed a significant interaction between maternal prepregnancy BMI and sex on total hippocampal volume (*p *< .001) such that boys (*r* = −.39, *p* = .018) but not girls (*r* = .11, *p* = .45) had a significant negative relationship between maternal prepregnancy BMI and total hippocampal volume. This relationship in boys remained significant after adjusting for child and maternal covariates (*β* = −126.98, *p* = .012). The sex interactions with prepregnancy BMI were consistently observed in hippocampal subfields CA1 (*p* = .008), CA2/3 (*p* = .016), CA4 (*p* = .002), dentate gyrus (*p* < .001), and subiculum (*p* < .001).

**Conclusions:**

Our results support findings in animal models and suggest that boys may be more vulnerable to the adverse effects of exposure to maternal obesity on hippocampal development than girls.

## Graphical abstract

We provide novel evidence in support of findings in rodent models suggesting that in utero exposure to maternal obesity differentially impacts the hippocampus of boys compared to girls during childhood. This is the first study in humans to show changes to the hippocampus in association with in utero exposure to maternal obesity.

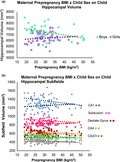

## SIGNIFICANCE

The hippocampus is sensitive to changes in the prenatal environment and is a brain region that is important for many aspects of learning, memory, and emotion regulation (Dimsdale‐Zucker, Ritchey, Ekstrom, Yonelinas, & Ranganath, 2018; Hayes et al., 2010; Herting & Nagel, 2012). The large pyramidal neurons within the hippocampus have a particularly high metabolic demand, which contribute to its high vulnerability to damage from metabolic and environmental perturbations (Hsu & Kanoski, 2014). Evidence from animal studies suggests that exposure to maternal obesity is linked to abnormal hippocampal development, with males being more impacted than females. This is the first human neuroimaging study to examine sex differences in relationships between exposure to maternal obesity and hippocampal volume in children. We found sex‐specific effects such that boys but not girls showed significant associations between prenatal exposure to maternal obesity and reductions in hippocampal volume. Sex differences were consistently observed across hippocampal subfields.

## 1 INTRODUCTION

Over 65% of U.S. women of childbearing age are overweight or obese (Ogden, Carroll, Kit, & Flegal, 2014), and the prevalence of maternal obesity continues to rise (Hales, Fryar, Carroll, Freedman, & Ogden, 2018). One of the most concerning aspects of this rising epidemic is the long‐term consequences on the development and health of the offspring, creating a vicious cycle of worsening health across generations. It is well established that offspring exposed to maternal obesity in utero are at increased risk for the development of metabolic disorders, including obesity, insulin resistance, and type 2 diabetes (Bider‐Canfield et al., 2017; Dabelea et al., 2008; Eriksson, Sandboge, Salonen, Kajantie, & Osmond, 2014; Maftei et al., 2015). Additionally, an array of animal studies have revealed that exposure to maternal obesity also leads to abnormal brain development in offspring (Argente‐Arizón et al., 2018; Bilbo & Tsang, 2010; Dearden & Balthasar, 2014; Edlow et al., 2016; Niculescu & Lupu, 2009a; Tozuka et al., 2010; Tozuka, Wada, & Wada, 2009; White et al., 2009; Zhu et al., 2018). Notably, the hippocampus is vulnerable to metabolic perturbations and sensitive to changes in the in utero environment (Hami, Karimi, Haghir, Gholamin, & Sadr‐Nabavi, 2015; Hami, Kerachian, Karimi, Haghir, & Sadr‐Nabavi, 2016; Lotfi, Hami, Hosseini, Haghir, & Haghir, 2016; Niculescu & Lupu, 2009a; Tozuka et al., 2009, 2010). Studies in mice have shown that exposure to maternal obesity leads to reductions in hippocampal neurogenesis within the dentate gyrus (DG), a subfield of the hippocampus (Niculescu & Lupu, 2009a; Tozuka et al., 2009). Moreover, mice exposed to maternal obesity in utero had decreased levels of hippocampal brain‐derived neurotrophic factor (BDNF) and abnormal dendritic differentiation of new hippocampal neurons during early postnatal development (Tozuka et al., 2010). Interestingly, sex differences in effects of prenatal exposure to maternal obesity were detected with fatty acid metabolite alterations within the hippocampus detected in male but not female mice offspring (Zhu et al., 2018). These findings are consistent with a wealth of data in animal models demonstrating sex‐specific effects of developmental programming by prenatal stress or exposure to maternal diabetes on brain structure and function, particularly in the hippocampus (Argente‐Arizón et al., 2018; Dearden & Balthasar, 2014; Edlow et al., 2016; Zagron & Weinstock, 2006; Zuena et al., 2008). Current literature suggests that two potential mechanisms mediate the greater susceptibility to prenatal exposures in males compared to females. First, estrogen has been shown to have anti‐inflammatory properties and may reduce cytokine exposure as well as protect against fat accumulation in the brain of female fetuses specifically (Argente‐Arizón et al., 2018; Kim, Young, Grattan, & Jasoni, 2014; Tiwari‐Woodruff & Voskuhl, 2009; Zhu et al., 2018). Second, because an adverse in utero environment may result in altered epigenetic modifications to the X‐chromosome and could specifically impact X‐linked genes important for brain development, females may be more protected by having two X‐chromosomes (Cox et al., 2015; Glendining & Jasoni, 2019; Martin et al., 2019; Schellong et al., 2019).

Findings from animal studies motivated us to examine sex differences in effects of in utero exposure to maternal obesity on hippocampal development in children. In this study, eighty‐eight children with varying degree of exposure to maternal obesity completed a high‐resolution structural magnetic resonance imaging (MRI) scan. Total hippocampal volume and hippocampal subfield volumes were quantified using FreeSurfer 6.0. Based on findings in rodent models (Argente‐Arizón et al., 2018; Dearden & Balthasar, 2014; Edlow et al., 2016; Zhu et al., 2018), we hypothesized that prenatal exposure to maternal obesity would be associated with greater hippocampal volume *reductions* in boys than girls. Moreover, we predicted that the DG would be particularly impacted by intrauterine exposure to maternal obesity in boys versus girls (Kim & Park, 2018; Niculescu & Lupu, 2009b; Tozuka et al., 2009) and further explored if sex differences were consistent across hippocampal subfields.

## 2 METHODS

### 2.1 Participants

Children between the ages of 7–11 years old participated in the BrainChild Study of the impact of intrauterine exposure to metabolic disorders on brain pathways during childhood. Children were born at Kaiser Permanente Southern California (KPSC), a large healthcare organization that uses an integrated electronic medical record (EMR) system. KPSC member demographics are broadly representative of Southern California residents (Koebnick et al., 2012). Children were excluded if they were born to mothers diagnosed with diabetes pre‐existing pregnancy or if they had a history of neurological, psychiatric, metabolic or other significant medical disorders, including diabetes, and/or used medications known to alter metabolism (i.e., glucocorticoids), had contraindications to magnetic resonance imaging (MRI) (e.g., metal implants, claustrophobia), or had a history of premature birth (<37 weeks’ gestation). Each participating Institutional Review Board approved this study (University of Southern California (USC) #HS‐14‐00034, KPSC #10282). Participants’ parents gave written informed consent. Children provided written informed assent.

### 2.2 Exposure

Maternal prepregnancy BMI was calculated from maternal height and weight measurements closest to last menstrual period from the EMR.

### 2.3 In‐person visits

The study included two in‐person visits. Visit one occurred at the Clinical Research Unit of the USC Diabetes and Obesity Research Institute. During this visit, child's height was measured to the nearest 0.1 cm using a stadiometer and weight to the nearest 0.1 kg using a calibrated digital scale. BMI was calculated using the standard formula, weight (kg) divided by height (m^2^). BMI *z*‐scores and BMI percentiles (age and sex‐specific standard deviation scores) were determined based on Center for Disease Control (CDC) standards (“Defining Childhood Obesity | Overweight & Obesity | CDC,” 2018). Participants were given the option of having Tanner stage assessed by physical examination (Marshall & Tanner, 1969, 1970) and/or by a validated sex‐specific assessment questionnaire for children and parents, containing both illustrations and explanatory text (Rasmussen et al., 2015). Forty‐eight participants opted for both physical examination and questionnaire. Forty participants opted for self‐reported puberty status only. The correlation between Tanner staging assessed by physical examination and questionnaire was 0.84.

Visit two occurred at USC Dana and David Dornsife Neuroimaging Center and included a MRI scan of the brain.

### 2.4 MRI Methods

After a mock scanner training session, magnetic resonance imaging was performed using a Siemens MAGNETOM Prisma^fit^ 3‐Tesla MRI scanner (Siemens Medical Systems) with a 20‐channel phased array coil. The MRI session started with a localizer scan. A high‐resolution MRI scan was acquired using a T1‐weighted three‐dimensional magnetization prepared rapid gradient echo (MP‐RAGE) sequence with the parameters: 256 × 256 × 176‐matrix size with 1 × 1 × 1‐mm^3^ resolution; inversion time = 900 ms; repetition time (TR) = 1,950 ms; echo time (TE) = 2.26 ms; flip angle = 90°; and total scan duration was 4 min and 14 s.

### 2.5 MRI analysis

The T1 MP‐RAGE structural image was put into the automated segmentation software, FreeSurfer version 6.0 hippocampal module **(** http://surfer.nmr.mgh.harvard.edu/, **RRID:SCR_001847**) to examine total hippocampal gray matter volume and gray matter volume in the hippocampal subfields. The procedure uses Bayesian inference and a probabilistic atlas of the hippocampal formation based on manual delineations of subfields in ultra‐high‐resolution MRI scans (Iglesias et al., 2015). For a detailed overview of the processing steps, please see Iglesias et al. (2015). Manual quality check of automated hippocampal segmentation was performed for each participant following an existing protocol (Backhausen et al., 2016). The segmentation of the hippocampus was visually assessed by an individual trained in hippocampal neuroanatomy and then given a rating of “pass,” “pass on condition,” and “fail.” Images that failed to have defined landmarks due to motion artifacts or segmentation error were excluded. The corresponding output, FS60, was used. It is a hippocampal proper parcellation with no head/body subdivisions. Although twelve subfield volumes are generated by FreeSurfer 6.0, we only included subfields that have been shown to be preferentially affected by prenatal exposures (i.e., gestational diabetes, prenatal stress) including the CA1, CA2/3, CA4, DG (granule cell layer), and subiculum (Florian & Nunes, 2011; Golalipour, Kafshgiri, & Ghafari, 2012; Graf et al., 2014; Lotfi et al., 2016; Niculescu & Lupu, 2009a; Tozuka et al., 2009; Wang et al., 2015; Zhou et al., 2018; Zhu et al., 2004). Previous studies in children have used Freesurfer to segment the hippocampus and hippocampal subfields (Al‐Amin, Zinchenko, & Geyer, 2018; Tamnes et al., 2014). The raw volume data are included in the supplemental materials (Table S1).

### 2.6 Statistical analysis

We examined sex differences in relationships between exposure to maternal obesity in utero and hippocampal volume in children. Exposure to maternal obesity in utero was indexed by maternal prepregnancy BMI obtained from EMR. The primary outcome measures were child's bilateral hippocampal volume and the volume of each hippocampal subfield. Covariates to control for potential confounding were child age, BMI *z*‐score, total intracranial volume (ICV), gestational diabetes mellitus (GDM) exposure obtained from EMR (dummy coded), and socioeconomic status (SES), which was assessed using household income at birth, estimated based on census tract of residence and expressed as a continuous variable, and maternal education at birth, which was extracted from birth certificates in the EMR as a categorical variable with the following categories: “high school or some high school,” “some college,” and “college and posteducation.”

Left and right hippocampal volumes were combined because correlations between the left and right hippocampus were high (*r* = .91, unadjusted *p* < .001) and there was not a statistically significant difference between the left hippocampus and the right hippocampus using Student's *t* test (*t* = −1.50, *p* = .13). Additionally, relationships between maternal obesity and hippocampal volume exhibited a similar pattern between the left (correlation coefficient *r* = −.13, unadjusted *p* = .25) and right hippocampus (correlation coefficient *r* = −.14, unadjusted *p* = .19). First, linear regression was used to assess whether there was a significant interaction between child sex and maternal prepregnancy BMI in the relationship with total hippocampal volume and the hippocampal subfields. Based upon a significant interaction, multivariable linear regression was used separately in boys and girls to investigate the relationship between maternal prepregnancy BMI and child hippocampal volume and subfields. The linear regression models were unadjusted models (Model 1), and models adjusting for ICV (Model 2), ICV and child age (Model 3), and with additional adjustments for SES and exposure to maternal GDM (Model 4), and additionally, child BMI *z*‐score (Model 5). These covariates were previously shown to influence hippocampal volume and thus were added to the model to control for potential confounding (Bauer et al., 2015; Hair, Hanson, Wolfe, & Pollak, 2015; Jabès, Thomas, Langworthy, Georgieff, & Nelson, 2015; Krogsrud et al., 2014; Mestre et al., 2017; Uematsu et al., 2012). Since the majority (93%) of children were prepubertal (Tanner stage < 2), Tanner stage was not adjusted in the regression models. There was also no difference in Tanner stage categories between boys and girls (*χ* ^2 ^= 1.84, *p* = .40). SAS 9.4 statistical software (SAS Institute) was used for all statistical analyses. For total hippocampal volume, a significance level of *p* < .05 was used. To control for false discovery rate (FDR) in the multiple comparisons of subfields, a FDR method based on the Benjamini–Hochberg procedure was used to assess significance within each model (Benjamini & Hochberg, 1995). Each *p*‐value was ranked and compared to the critical value with an overall false discovery rate at 5%.

## 3 RESULTS

### 3.1 Participants’ characteristics

Of the 117 children enrolled into the study, 99 of these children completed MRI scans. Ten children were excluded due to excessive motion and one was excluded due to incidental brain findings leaving a total of 88 children in the MRI analyses (Figure S1) (Alves, 2019). The mean ± *SD* age was 8.4 ± 0.9 years old, 93% of the children were prepubertal (Tanner stage < 2), and 58.0% were girls (Table 1). Maternal prepregnancy BMI ranged from 19.0 to 50.4 kg/m^2^. Overall, 22.7% of mothers were normal weight, 36.4% were overweight, and 40.9% were obese prior to pregnancy. Children's BMI ranged from 13.6 to 34.0 kg/m^2^; BMI percentiles ranged from 5.3 to 99.6; and BMI *z*‐scores ranged from −1.78 to 2.64. Based on CDC standards, 61% of children were classified as healthy weight, 15% were classified as overweight, and 24% of children were classified as obese (“Defining Childhood Obesity | Overweight & Obesity | CDC,” 2018). The characteristics of the boys and girls were not significantly different (Table S2).

**Table 1:** Characteristic of the 88 child participants and their mothers

Child characteristics	Mean (*SD*) or *N* (%)	Range
Age, years	8.37 (0.89)	7.33 ~ 11.23
Body mass index (BMI), kg/m^2^	18.68 (3.97)	13.62 ~ 34.01
BMI percentile	68.62 (27.52)	5.28 ~ 99.58
BMI *z*‐score	0.73 (1.09)	−1.78 ~ 2.64
BMI category	Healthy weight: 54 (61%) Overweight: 13 (15%) Obese: 21 (24%)	
Sex	Boys: 37 (42%) Girls: 51 (58%)	
Tanner stage of pubertal development	Tanner stage 1:82 (93%) Tanner stage 2:5 (6%) Tanner stage 3:1 (1%)	
Maternal characteristics		
Maternal prepregnancy BMI, kg/m^2^	29.86 (6.90)	18.97 ~ 50.38
Maternal education	Missing: 2 (2%)^a^ ≤High school: 23 (26%)^a^ Some college: 17 (19%)^a^ College and post: 46 (52%)^a^	
Family income	Missing: 2 (2%) 0 ≤ income < 30,000:7 (8%) 30,000 ≤ income < 50,000:22 (25%) 50,000 ≤ income < 70,000:30 (34%) 70,000 ≤ income < 90,000:14 (16%) 90,000 ≤ income: 13 (15%)	
Mother's race/ethnicity	Hispanic: 49 (56%) Black: 10 (11%) Non‐Hispanic White: 19 (22%) Other: 10 (11%)	

a Percentages were rounded to the nearest percent and therefore may not equal to 100%.

### 3.2 Interaction between maternal prepregnancy BMI and sex on child hippocampal volume

**Figure 1:**
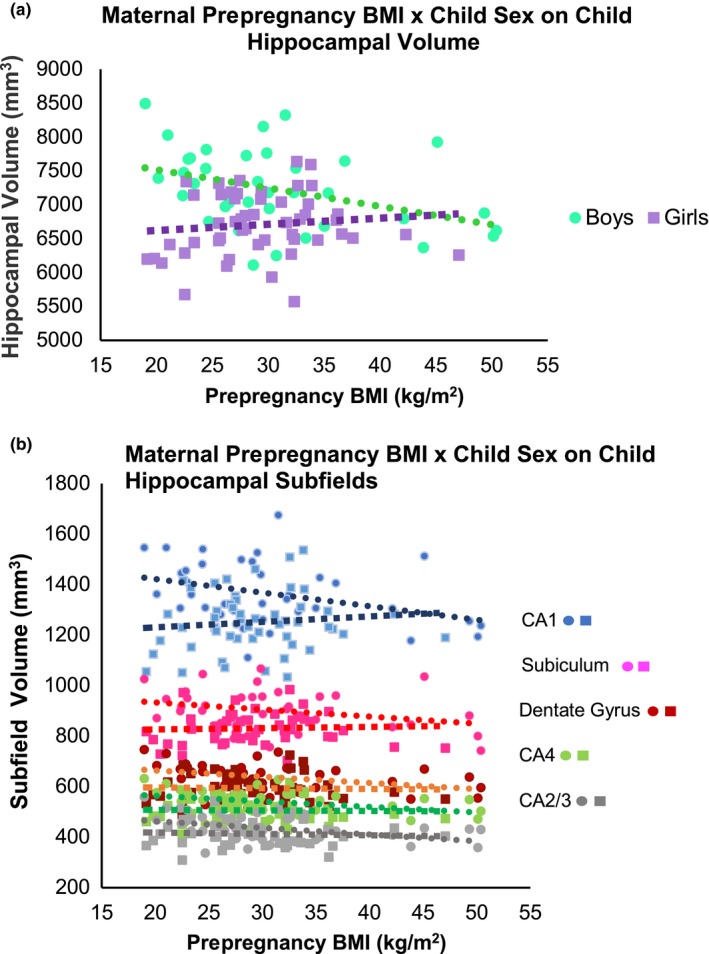
For (a), boys depicted as “turquoise circles.” Girls depicted as “purple squares”; for (b), boys depicted as “circles.” Girls depicted as “squares.” CA1 subfield denoted in blue, subiculum denoted in pink, dentate gyrus denoted in red, CA4 denoted in green, and CA2/3 denoted in gray

A significant interaction between prepregnancy BMI and sex on child hippocampal volume was observed in unadjusted model (*p* < .001) as well as after adjusting for child and maternal covariates including child intracranial volume (ICV), age, socioeconomic status (SES), maternal gestational diabetes mellitus (GDM) status, and child BMI *z*‐score (*p* = .024). Figure 1a depicts the scatter plot of the data for boys and girls. When stratified by sex, a negative relationship between maternal prepregnancy BMI and hippocampal volume was observed in boys (correlation coefficient *r* = −.39, *p* = .018) but not in girls (*r* = .11, *p* = .45). The significant negative relationship in boys remained after adjusting for child and maternal covariates (*β* = −126.98, *SE* = 47.26, *p* = .01; Table 2).

**Table 2:** Regression coefficients between maternal prepregnancy BMI per 5 unit increments and total hippocampal volume and hippocampal subfield volumes in boys, (*N* = 37)

Region	Model 1	Model 2	Model 3	Model 4	Model 5
Hippocampus					
*β* (*SE*)	−134.97 (54.35)	−103.84 (41.94)	−103.55 (42.60)	−113.05 (44.26)	−126.98 (47.26)
*p*‐value	.018*	.018*	.021*	.016*	.012*
CA1					
*β* (*SE*)	−26.88 (11.83)	−21.67 (10.46)	−21.19 (10.46)	−21.57 (10.87)	−23.58 (11.71)
*p*‐value	.029^a^	.046	.051	.057	.054
CA2/3					
*β* (*SE*)	−13.20 (4.82)	−11.05 (4.25)	−11.26 (4.24)	−12.44 (4.47)	−14.19 (4.73)
*p*‐value	.010^a^	.014	.012	.009^a^	.006^a^
CA4					
*β* (*SE*)	−10.98 (4.67)	−8.38 (3.68)	−8.55 (3.68)	−9.63 (3.92)	−10.34 (4.22)
*p*‐value	.025^a^	.029	.026	.020^a^	.021^a^
Dentate gyrus					
*β* (*SE*)	−12.30 (5.44)	−9.16 (4.17)	−9.29 (4.21)	−10.36 (4.52)	−11.31 (4.86)
*p*‐value	.030^a^	.035	.034	.029^a^	.027^a^
Subiculum					
*β* (*SE*)	−13.98 (7.76)	−10.49 (6.81)	−10.17 (6.80)	−11.80 (7.01)	−12.51 (7.58)
*p*‐value	.080	.13	.14	.10	.11

Notes

Model 1: unadjusted.

Model 2: adjusted for ICV.

Model 3: adjusted for ICV + child age.

Model 4: adjusted for ICV + child age + SES +maternal gestational diabetes mellitus (GDM) status.

Model 5: adjusted for ICV + child age + SES +maternal GDM status + BMI *z*‐score.

a Denotes significance remained after FDR correction for multiple subfields at a threshold of *q* = 0.05.

* Significance level at *p* < .05.

Significant interactions were also observed in the CA1 (*p* = .008), CA2/3 (*p* = .016), CA4 (*p* = .002), DG (*p* < .001), and subiculum (*p* < .001) hippocampal subfields, respectively (unadjusted interaction test); all remained significant after applying Benjamini–Hochberg procedure to control for false discovery rate (FDR) among the multiple subfields. Figure 1b depicts the scatter plot of the data for boys and girls in each subfield. When stratified by sex, a negative relationship between maternal prepregnancy BMI and the volume of CA1, CA2/3, CA4, and DG hippocampal subfields was observed in boys (CA1: *r* = −.36, *p* = .029; CA2/3: *r* = −.42, *p* = .010; CA4: *r* = −.37, *p* = .025; DG: *r* = −.36, *p* = .030) (Figure 1b; Table 2) but not in girls (CA1: *r* = .11, *p* = .47; CA2/3: *r* = −.06, *p* = .66; CA4: *r* = −.03, *p* = .84; DG: *r* = −.03, *p* = .82) (Figure 1b; Table 3). After adjusting child and maternal covariates, the negative relationship in boys remained significant in the CA2/3 (*β* = −14.19, *SE* = 4.73, *p* = .006), CA4 (*β* = −10.34, *SE* = 4.22, *p* = .021), and DG (*β* = −11.31, *SE* = 4.86, *p* = .027) subfields (Table 2); all survived FDR correction.

**Table 3:** Regression coefficients between maternal prepregnancy BMI per 5 unit increments and total hippocampal volume and hippocampal subfield volumes in girls, (*N* = 51)

Region	Model 1	Model 2	Model 3	Model 4	Model 5
Hippocampus					
*β* (*SE*)	45.51 (59.31)	54.26 (47.96)	51.66 (49.12)	61.39 (52.33)	28.78 (55.35)
*p*‐value	.45	.26	.30	.25	.61
CA1					
*β* (*SE*)	10.83 (14.95)	13.07 (11.96)	11.54 (12.19)	14.37 (13.01)	7.56 (13.89)
*p*‐value	.49	.28	.35	.28	.59
CA2/3					
*β* (*SE*)	−2.53 (6.03)	−1.88 (5.11)	−0.37 (5.08)	0.25 (5.46)	−1.44 (5.91)
*p*‐value	.68	.72	.94	.96	.81
CA4					
*β* (*SE*)	−1.04 (5.43)	−0.39 (4.72)	0.01 (4.83)	0.55 (4.98)	−1.59 (5.35)
*p*‐value	.85	.93	>.99	.91	.77
Dentate gyrus					
*β* (*SE*)	−1.53 (6.51)	−0.61 (5.59)	−0.50 (5.73)	0.40 (5.95)	−2.53 (6.37)
*p*‐value	.82	.91	.93	.95	.69
Subiculum					
*β* (*SE*)	2.72 (7.76)	3.30 (7.16)	2.35 (7.29)	2.97 (7.90)	−0.61 (8.48)
*p*‐value	.74	.65	.75	.71	.94

Notes

Model 1: unadjusted.

Model 2: adjusted for ICV.

Model 3: adjusted for ICV + child age.

Model 4: adjusted for ICV + child age + SES +maternal gestational diabetes mellitus (GDM) status.

Model 5: adjusted for ICV + child age + SES + maternal GDM status + BMI *z*‐score.

In unadjusted and unstratified analyses, maternal prepregnancy BMI was not significantly associated with child's hippocampal volume (*β* = −46.97, *SE* = 44.85, *p* = .30) (Table S3). However, in models adjusted for child ICV, age, sex, and the interaction between maternal prepregnancy BMI and child sex, we observed a significant association between maternal prepregnancy BMI and total hippocampal volume in children (*β* = 106.98, *SE* = 39.40, *p* = .008).

## 4 DISCUSSION

In this study, we investigated sex‐specific effects of in utero exposure to obesity on hippocampal volume in children aged 7–11 years. Consistent with results from animal models, we observed a significant interaction of prepregnancy BMI and sex on hippocampal volume, such that boys but not girls showed a significant negative relationship between prepregnancy BMI and hippocampal volume. These sex‐specific effects were consistently observed in the hippocampal subfields. Our findings suggest that boys may be more vulnerable to maternal obesity induced altered hippocampal development than girls.

A number of animal studies have revealed that prenatal exposure to maternal obesity is linked to abnormal hippocampal development, including reductions in neurogenesis, decreased levels of BDNF (a neurotrophin involved in neural differential and survival), and abnormal dendritic differentiation of new neurons (Niculescu & Lupu, 2009b; Tozuka et al., 2009, 2010). Several lines of evidence in rodent models suggest potential mechanisms by which maternal obesity influences the development of the fetal hippocampus. First, exposure to maternal obesity may elevate levels of inflammation in the fetus, and the hippocampus is vulnerable to a pro‐inflammatory environment (Bilbo & Tsang, 2010; Graf et al., 2014; White et al., 2009). Prenatal exposure to an aberrant inflammatory environment is associated with increased microglial cells and inflammatory markers (i.e., toll‐like receptor 4) within the hippocampus of offspring (Bilbo & Tsang, 2010; Graf et al., 2014; Ornellas, Mello, Mandarim‐de‐Lacerda, & Aguila, 2013; White et al., 2009). Second, maternal obesity exposes the fetus to an excessive nutrient supply, which may in turn stimulate fetal hyperinsulinemia (Lecoutre et al., 2016; Murabayashi et al., 2013). Fetal hyperinsulinemia has been shown to alter hippocampal development via impaired insulin signaling and reduced neurogenesis in the hippocampus (Schmitz et al., 2018). Notably, insulin signaling in the hippocampus is important for both neurogenesis and learning and memory (Benedict, 2004; Hui, Pei, Zhang, Guan, & Zhang, 2005; Kern et al., 2001; Park, Seeley, Craft, & Woods, 2000).

Interestingly, prior evidence suggests that the brain development of males may be more susceptible to metabolic and environmental perturbations encountered in utero when compared to female offspring (Argente‐Arizón et al., 2018; Dearden & Balthasar, 2014; Schulz et al., 2011; Zuena et al., 2008). For example, prenatal exposure to maternal obesity resulted in alterations in fatty acid metabolites within the hippocampus of male but not female offspring (Zhu et al., 2018). Studies investigating the role of prenatal exposures to stress and alcohol have also found sex differences in the vulnerability of the hippocampus to alterations in the intrauterine environment, with more pronounced effects in males than females (Schulz et al., 2011; Treit et al., 2017; Zuena et al., 2008). Moreover, male offspring exposed to obese mothers in utero were found to have abnormal gene expression within the hypothalamus and forebrain, whereas female offspring did not (Dearden & Balthasar, 2014). Motivated by these compelling findings in animal studies (Argente‐Arizón et al., 2018; Dearden & Balthasar, 2014; Edlow et al., 2016; Zhu et al., 2018), we examined for the first time in humans the sex‐specific effects of exposure to maternal obesity in utero on hippocampal development in children.

In vivo structural neuroimaging provides a noninvasive methodology for investigating neuronal volume indirectly via gray matter volume quantification (Pohlack et al., 2014). Prior studies have used structural MRI to measure hippocampal volume in pediatric populations (Barnea‐Goraly et al., 2014; Bauer et al., 2015; Hershey et al., 2010; Jabès et al., 2015; Mestre et al., 2017; Plessen et al., 2006) and found reduced hippocampal volume in children with diabetes and metabolic syndrome (Bruehl, Sweat, Tirsi, Shah, & Convit, 2011; Yau, Castro, Tagani, Tsui, & Convit, 2012). Here, we used structural MRI methods to examine the effects of prenatal exposure to maternal obesity on hippocampal volume in children. While unadjusted and unstratified analyses did not show significantly significant associations between maternal prepregnancy BMI and child's hippocampal volume, in models adjusted for child ICV, age, sex, and the interaction between maternal prepregnancy BMI and child sex, we observed a significant association between maternal prepregnancy BMI and total hippocampal volume in children. Moreover, we observed a significant interaction between maternal prepregnancy BMI and sex on child's hippocampal volume, and in analyses stratified by sex, we showed that boys but not girls had a significant negative association between maternal prepregnancy BMI and total hippocampal volume. Adjusting for confounding variables did not affect these results. Sex differences were also found in the hippocampal subfields CA1, CA2/3, CA4, DG, and subiculum. These results are consistent with findings in rodent models and provide additional evidence that boys may be particularly susceptible to the altered in utero environment characteristic of maternal obesity during pregnancy.

Our results are also in line with prior studies in humans that have observed sex differences in the effects of prenatal exposures on brain development (de Rooij et al., 2016; Treit et al., 2017). Evans and Myatt (2017) showed that male fetuses of obese mothers have higher levels of reactive oxidative species compared to female fetuses (Evans & Myatt, 2017). It is possible that girls may be more protected by adverse events in utero due to the anti‐inflammatory and neuroprotective properties of estrogen (Gillies, Murray, Dexter, & McArthur, 2004; Shivers et al., 2015; Toung, Traystman, & Hurn, 1998). Interestingly, boys are also more likely to be diagnosed with neurodevelopmental disorders such as autism spectrum disorder (ASD) and attention deficit hyperactivity disorder (ADHD) (Gillberg, Cederlund, Lamberg, & Zeijlon, 2006; Li et al., 2016), and epidemiological studies have found that maternal obesity is associated with an increased risk for developing ASD and ADHD (Krakowiak et al., 2012; Li et al., 2016; Musser et al., 2017). One study found that children diagnosed with ASD or ADHD were not only more likely to be male but were also more likely to have been exposed to maternal obesity in utero (Li et al., 2016). It is possible that the observed sex‐specific effects of prenatal exposure to maternal obesity on hippocampal development in our study could be associated with increased risk of neurodevelopmental disorders in boys compared with girls; however, future studies are needed to address this possibility.

Collectively, our findings suggest that the hippocampus is vulnerable to metabolic insults and that structural MRI is a sensitive method to detect changes in hippocampal volume in relation to metabolic disorders.

### 4.1 Limitations

While our study investigated relationships between maternal obesity and hippocampal volume, it remains to be determined whether these structural alterations are also associated with functional deficits. Future work should investigate the functional implications of reduced hippocampal volume by examining whether reduced hippocampal volume is associated with cognitive or behavioral impairments in offspring exposed prenatally to maternal obesity. We were unable to investigate potential underlying mechanisms by which maternal obesity affects fetal hippocampal development due to the current structure of study design, and these should be addressed in future studies. Further, due to the cross‐sectional nature of this study, it is unknown whether the relationship observed predominately in boys will persist throughout life. Because the growth trajectory for peak hippocampal volume in girls can occur at an earlier age than boys, it is possible that we are observing a delay in brain maturation in boys and missed the relationship in girls (Krogsrud et al., 2014; Lenroot & Giedd, 2006). Future studies should consider a longitudinal assessment to determine whether this relationship is due to delayed maturation or whether reduced hippocampal volume persists among children exposed to maternal obesity. Additionally, although manual tracing is considered the gold standard for hippocampal segmentation, recent advances in automated segmentation methods, such as the newly developed Freesurfer 6.0, provide a rigorous and practical method to quantify hippocampal volume overall and within each subfield (Cover, van Schijndel, Bosco, Damangir, & Redolfi, 2018; Schmidt et al., 2018; Whelan et al., 2016). Freesurfer 6.0 has increased accuracy and reliability making it comparable to manual segmentation while being more efficient (Cover et al., 2018; Iglesias et al., 2015; Schmidt et al., 2018; Whelan et al., 2016). However, the anatomically defined boundaries created by Freesurfer 6.0’s ultra‐high‐resolution scan may be more precise than captured by our high‐resolution T1 image.

## 5 CONCLUSION

In summary, we found a significant interaction of maternal prepregnancy BMI and sex on child hippocampal volume. Boys but not girls showed a significant negative correlation between prepregnancy BMI and hippocampal volume, suggesting that boys may be more vulnerable than girls to the adverse effects of exposure to maternal obesity on hippocampal development. These results call for more attention to considering sex differences on the effects of prenatal exposure to maternal obesity on brain and cognitive development during childhood. Additionally, given the important role of the hippocampus in various cognitive functions, this study provides a potential neurobiological underpinning into the link between maternal obesity and the previously noted cognitive deficits and neurodevelopmental disorders observed in offspring.

## Data Availability

The imaging data that support the findings of this study are openly available in Open Science Framework at https://osf.io/egdk8/, doi.10.17605/OSF.IO/EGDK8. Additional data that support the findings of this study are available on request from the corresponding author. The data are not publicly available due to privacy or ethical restrictions.
