# Supplementary material for: Sex differences in the association between prenatal exposure to maternal obesity and hippocampal volume in children
Source: Brain Behav. 2020 Jan 5;10(2):e01522. doi: 10.1002/brb3.1522 (PMC7010582; doi:10.1002/brb3.1522)
Supplement: Supplementary file 1 [file BRB3-10-e01522-s001.docx]

# Supplemental Information

SI Figure 1 Recruitment Flowchart of BrainChild Study

SI Table 1. Mean Total Measurements of Intracranial volume, Hippocampal Volume and the Hippocampal Subfields.

|  | Mean (SD) | Range |
| --- | --- | --- |
| Intracranial Volume (mm^3^) | 1 410 871.5 (120930.3) | 1140714.9~1656738 |
| Hippocampal Volume (mm^3^) | 6924.7 (578.4) | 5575.8~8493.9 |
| CA1 Volume (mm^3^) | 1298.5 (132.9) | 1034.6~1674.4 |
| CA2/3 Volume (mm^3^) | 422.5 (50.5) | 309~559 |
| CA4 Volume (mm^3^) | 520.5 (48.5) | 409.6~632.7 |
| Dentate Gyrus Volume (mm^3^) | 612.4 (57.8) | 490.2~747.8 |
| Subiculum (mm^3^) | 861.5 (78) | 702.5~1067.1 |

SI Table 2. Summary of Child Participants (37 boys, 51 girls) and their Mothers Stratified by Sex.

| **Child Characteristics** | **Boys: Mean (SD) or N (%)** | **Girls: Mean (SD) or N (%)** | **P-value** |
| --- | --- | --- | --- |
| Age, years | 8.38 (1.01) | 8.35 (0.80) | 0.86 |
| Body mass index (BMI), kg/m^2^ | 18.68 (3.78) | 18.68 (4.14) | >0.99 |
| BMI percentile | 68.83 (29.75) | 68.48 (26.08) | 0.95 |
| BMI z-score | 0.76 (1.17) | 0.70 (1.04) | 0.82 |
| BMI category | Healthy-weight: 20 (54%)  Overweight: 6 (16%)  Obese: 11 (30%) | Healthy-weight: 34 (67%)  Overweight: 7 (14%)  Obese: 10 (19%) | 0.46 |
| Sex | Boys: 37 (42%)  Girls: 51 (58%) | |  |
| Tanner Stage of Pubertal Development | Tanner stage 1: 36 (97%)  Tanner stage 2: 1 (3%)  Tanner stage 3: 0 (0%) | Tanner stage 1: 46 (90%)  Tanner stage 2: 4 (8%)  Tanner stage 3: 1 (2%) | 0.50 |
| **Maternal Characteristics** | | | |
| Maternal pre-pregnancy BMI, kg/m^2^ | 30.70 (8.44) | 29.25 (5.55) | 0.37 |
| Maternal education | Missing:  <=High school: 10 (27%)  Some college: 7 (19%)  College and post: 20 (54%) | Missing: 2 (4%)  <=High school: 13 (25%)  Some college: 10 (20%)  College and post: 26 (51%) | 0.52 |
| Family income | \| Missing: \| \| --- \| \| 0<=income <30 000: 4 (11%) \| \| 30000<=income <50 000: 6 (16%) \| \| 50000<=income <70 000: 13 (35%) \| \| 70000<=income <90 000: 7 (19%) \| \| 90000>=income: 7 (19%) \| | \| Missing: 2 (4%) \| \| --- \| \| 0<=income <30 000: 3 (6%) \| \| 30000<=income <50 000: 16 (31%) \| \| 50000<=income <70 000: 17 (33%) \| \| 70000<=income <90 000: 7 (14%) \| \| 90000>=income: 6 (12%) \| | 0.42 |
| Mother’s race/ethnicity | Hispanic: 20 (54%)  Black: 2 (5%)  Non-Hispanic White: 11 (30%)  Other: 4 (11%) | Hispanic: 29 (57%)  Black: 8 (16%)  Non-Hispanic White: 8 (16%)  Other: 6 (12%) | 0.27 |

^a^Student’s t-tests, chi-square tests and Fisher’s exact tests were used to test for group differences.

SI Table 3. Relationships between Maternal Pre-pregnancy BMI (5 unit increments) and Total Hippocampal Volume and Hippocampal Subfield Volume (N=88).

| **Region** |  |  | | | | |
| --- | --- | --- | --- | --- | --- | --- |
| **Hippocampus** | **Model 1** | **Model 2** | **Model 3** | **Model 4** | **Model 5** | **Model 6** |
| Beta (SE) | -46.97 (44.85) | -41.15 (31.62) | -46.14 (32.27) | -106.98 (39.40) | -113.52 (40.76) | -130.13 (40.87) |
| P-value | 0.30 | 0.20 | 0.16 | 0.008* | 0.007* | 0.002* |
| **CA1** | **Model 1** | **Model 2** | **Model 3** | **Model 4** | **Model 5** | **Model 6** |
| Beta (SE) | -8.34 (10.34) | -7.10 (7.79) | -8.14 (7.95) | -20.86 (9.81) | -21.11 (10.18) | -24.32 (10.38) |
| P-value | 0.42 | 0.37 | 0.31 | 0.037^a^ | 0.036^a^ | 0.022^a^ |
| **CA2/3** | **Model 1** | **Model 2** | **Model 3** | **Model 4** | **Model 5** | **Model 6** |
| Beta (SE) | -8.34 (3.84) | -7.94 (3.19) | -7.47 (3.26) | -10.89 (4.09) | -11.97 (4.21) | -13.10 (4.29) |
| P-value | 0.033 | 0.015 | 0.025 | 0.009^a^ | 0.006^a^ | 0.003^a^ |
| **CA4** | **Model 1** | **Model 2** | **Model 3** | **Model 4** | **Model 5** | **Model 6** |
| Beta (SE) | -5.90 (3.73) | -5.46 (2.87) | -5.65 (2.94) | -8.78 (3.69) | -9.66 (3.85) | -10.82 (3.90) |
| P-value | 0.12 | 0.060 | 0.058 | 0.020^a^ | 0.014^a^ | 0.007^a^ |
| **Dentate Gyrus** | **Model 1** | **Model 2** | **Model 3** | **Model 4** | **Model 5** | **Model 6** |
| Beta (SE) | -6.51 (4.46) | -5.97 (3.33) | -6.26 (3.42) | -9.61 (4.29) | -10.48 (4.47) | -11.99 (4.52) |
| P-value | 0.15 | 0.077 | 0.071 | 0.028^a^ | 0.022^a^ | 0.010^a^ |
| **Subiculum** | **Model 1** | **Model 2** | **Model 3** | **Model 4** | **Model 5** | **Model 6** |
| Beta (SE) | 5.00 (6.07) | -4.34 (4.89) | -5.38 (4.90) | -11.00 (6.13) | -11.71 (6.25) | -13.37 (6.36) |
| P-value | 0.41 | 0.38 | 0.28 | 0.077 | 0.065 | 0.039^a^ |

Model 1: unadjusted

Model 2: adjusted for ICV

Model 3: adjusted for ICV + child age + sex

Model 4: adjusted for ICV + child age + sex + interaction of pre-pregnancy BMI and sex

Model 5: adjusted for ICV + child age + sex + interaction of pre-pregnancy BMI and sex + SES + GDM

Model 6: adjusted for ICV + child age + sex + interaction of pre-pregnancy BMI and sex + SES + GDM + BMI z-score

*Denotes a significance level at P<0.05.

^a^Denotes significance remained after FDR was used for multiple comparisons within each model.
